# Development and Selection of the Human Vγ9Vδ2^+^ T-Cell Repertoire

**DOI:** 10.3389/fimmu.2018.01501

**Published:** 2018-07-02

**Authors:** Carrie R. Willcox, Martin S. Davey, Benjamin E. Willcox

**Affiliations:** Cancer Immunology and Immunotherapy Centre, Institute for Immunology and Immunotherapy, University of Birmingham, Birmingham, United Kingdom

**Keywords:** gamma/delta T-cell, T-cell receptor repertoire, Vγ9Vδ2^+^ T-cell, phosphoantigen, HMBPP

## Abstract

Vγ9Vδ2^+^ lymphocytes are among the first T-cells to develop in the human fetus and are the predominant peripheral blood γδ T-cell population in most adults. Capable of broad polyclonal responses to pyrophosphate antigens (pAg), they are implicated in immunity to a diverse range of infections. Previously Vγ9Vδ2^+^ development was thought to involve postnatal selection and amplification of public Vγ9 clonotypes in response to microbial stimuli. However, recent data indicate the Vγ9Vδ2^+^ T-cell receptor (TCR) repertoire, which is generated early in gestation, is dominated by public Vγ9 clonotypes from birth. These chains bear highly distinct features compared to Vγ9 chains from Vδ1^+^ T-cells, due either to temporal differences in recombination of each subset and/or potentially prenatal selection of pAg-reactive clonotypes. While these processes result in a semi-invariant repertoire featuring Vγ9 sequences preconfigured for pAg recognition, alterations in TCRδ repertoires between neonate and adult suggest either peripheral selection of clonotypes responsive to microbial antigens or altered postnatal thymic output of Vγ9Vδ2^+^ T-cells. Interestingly, some individuals demonstrate private Vγ9Vδ2^+^ expansions with distinct effector phenotypes, suggestive of selective expansion in response to microbial stimulation. The Vγ9Vδ2^+^ T-cell subset, therefore, exhibits many features common to mouse γδ T-cell subsets, including early development, a semi-invariant TCR repertoire, and a reliance on butyrophilin-like molecules in antigen recognition. However, importantly Vγ9Vδ2^+^ T-cells retain TCR sensitivity after acquiring an effector phenotype. We outline a model for Vγ9Vδ2^+^ T-cell development and selection involving innate prenatal repertoire focusing, followed by postnatal repertoire shifts driven by microbial infection and/or altered thymic output.

## Development of the Vγ9Vδ2^+^ T-Cell Compartment

Vγ9Vδ2^+^ lymphocytes are the predominant γδ T-cell subset in healthy adult peripheral blood. Essentially all Vγ9Vδ2^+^ T-cells respond to small pyrophosphate antigens (pAg) (1) in a T-cell receptor (TCR)-dependent manner (2), a process dependent on target cell expression of the butyrophilin (BTN) family member BTN3A1 (3). The population expands during childhood (4), typically comprising ~1–10% of total peripheral blood T-cells in healthy adults.

**Figure 1:**
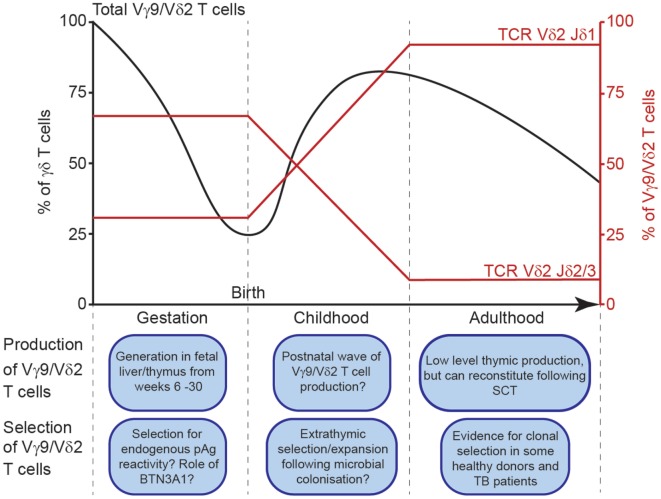
Schematic depiction of Vγ9Vδ2^+^ T cell generation and selection throughout life. Vγ9Vδ2^+^ T cells as a percentage of total peripheral blood γδ T cells throughout life (black line, left axis). Jδ usage among Vγ9Vδ2^+^ T cells (red lines, right axis) throughout life.

The Vγ9 and Vδ2 variable (V) gene segments are the first γ/δ chains to undergo rearrangement in development, detected in fetal liver from as early as 5–6 weeks gestation (5), and in fetal thymus after 8 weeks gestation (6). By mid-gestation (20–30 weeks), Vγ9Vδ2^+^ T-cells dominate the γδ repertoire (7) (Figure 1). However, Vδ1^+^ T-cell generation increases later in gestation, and Vδ1^+^ T-cells comprise the majority of the γδ repertoire in cord blood (7, 8), and in pediatric thymus (9). It is unclear whether gestationally produced Vγ9Vδ2^+^ cells persist in fetal blood, and become outnumbered by subsequent Vδ1^+^ T-cell production, or whether most Vγ9Vδ2^+^ T-cells exit circulation and populate the tissues. However, the dramatic postnatal numerical expansion of Vγ9Vδ2^+^ T-cells likely occurs following microbial exposure, with the Vγ9Vδ2^+^ subset ultimately dominating the circulating γδ T-cell repertoire during childhood (4, 10). Consistent with this, Vγ9Vδ2^+^ T-cells mature in phenotype early after birth concomitant with their numerical expansion (4); moreover, several infections stimulate Vγ9Vδ2^+^ expansion, and tellingly, identical twins have different Vγ9Vδ2^+^ profiles (4).

## The Vγ9Vδ2^+^ TCR Repertoire in Healthy Adults

Early studies identified Vγ9Vδ2^+^ TCR features required for pAg responsiveness. Interestingly, adult Vδ2 CDR3s were highly diverse, composed of Vδ2 joined to one (or occasionally two) diversity (D) segments (usually Dδ3), and typically used joining (J) segment Jδ1 (11, 12). A hydrophobic amino acid, typically Val/Leu/Ile at position 97 of the Vδ2 framework (position 5 of the CDR3, defined as the amino acids between the Vδ2 segment C-terminal Cys and the conserved Phe of the J segment), generated by N-nucleotide addition, was required for pAg recognition (12, 13).

Conversely, Vγ9 gene segments were relatively restricted in CDR3γ sequence and length, and exclusively utilized JγP and constant region Cγ1 (11, 14, 15). One clonotype (CALW**EVQ**ELGKKIKVF), generated by germline Vγ9-JγP recombination with minimal nucleotide trimming and no N-nucleotide addition, was present in many healthy donors (15). Further low-throughput analyses detected many “public” Vγ9 clonotypes in multiple individuals (16). Although peripheral blood γδ T-cell numbers vary widely between individuals and are influenced by age and sex (17), public clonotypes are conserved irrespective of age, sex, and race (16), and between cord blood and adult (18). Although the presence of such public Vγ9 sequences was thought to reflect strong postnatal peripheral selection and amplification of specific clonotypes following microbial exposure (19), an improved understanding of the Vγ9Vδ2^+^ TCR repertoire suggests alternative possibilities.

## Evidence for Convergent Recombination in the Vγ9 TCR Repertoire

Deep sequencing analyses of Vγ9Vδ2^+^ TCR repertoires (20–23) have confirmed a high frequency of public Vγ9 clonotypes in adult Vγ9Vδ2^+^ T-cells, and reveal the basis for Vγ9 TCR publicity. The most prevalent of these, CALW**EVQ**ELGKKIKVF, highlighted in many previous studies (7, 11, 15, 16, 18), comprised between 4 and 45% of the Vγ9 repertoire (20–22). As noted (15), this amino acid sequence can be generated by near-germline recombination of Vγ9 and JγP gene segments with minimal nucleotide trimming and no N-nucleotide addition. However, it can also result from several different nucleotide sequences: (1) involving trimming of nucleotides at the 3′ end of the V region and/or 5′ end of the J region, (2) incorporation of one or more palindromic (P)-nucleotides, and/or (3) addition of one or several non-templated (N)-nucleotides by terminal deoxynucleotide transferase (TdT), resulting in the same amino acid sequence (Table 1). Moreover, other public Vγ9 clonotypes can be generated in multiple ways depending on the extent of V and J gene segment trimming, and N/P-nucleotide addition (Table 1) (23).

**Table 1:** Common public Vγ9-JγP sequences can be generated by convergent recombination.

	Vγ9						P	N	P		JγP											P nt	N nt

Germline	TGT	GCC	TTG	TGG	GAG	GTG				T	GGG	CAA	GAG	TTG	GGC	AAA	AAA	ATC	AAG	GTA	TTT		
CALW**EVQ**ELGKKIKVF																							
	TGT	GCC	TTG	TGG	GAG	GTG						CAA	GAG	TTG	GGC	AAA	AAA	ATC	AAG	GTA	TTT	0	0
	TGT	GCC	TTG	TGG	GAG	GT		C				CAA	GAG	TTG	GGC	AAA	AAA	ATC	AAG	GTA	TTT	0	1
	TGT	GCC	TTG	TGG	GAG	GT		A				CAA	GAG	TTG	GGC	AAA	AAA	ATC	AAG	GTA	TTT	0	1
	TGT	GCC	TTG	TGG	GAG	GT		T				CAA	GAG	TTG	GGC	AAA	AAA	ATC	AAG	GTA	TTT	0	1
	TGT	GCC	TTG	TGG	GAG	GTG	CA	G					GAG	TTG	GGC	AAA	AAA	ATC	AAG	GTA	TTT	2	1

CALW**EVR**ELGKKIKVF																							
	TGT	GCC	TTG	TGG	GAG	GTG	C	G				A	GAG	TTG	GGC	AAA	AAA	ATC	AAG	GTA	TTT	1	1
	TGT	GCC	TTG	TGG	GAG	GTG		AG				A	GAG	TTG	GGC	AAA	AAA	ATC	AAG	GTA	TTT	0	2
	TGT	GCC	TTG	TGG	GAG	GTG	C	GT					GAG	TTG	GGC	AAA	AAA	ATC	AAG	GTA	TTT	1	2
	TGT	GCC	TTG	TGG	GAG	GTG	C	GC					GAG	TTG	GGC	AAA	AAA	ATC	AAG	GTA	TTT	1	2
	TGT	GCC	TTG	TGG	GAG	GTG	C	GG					GAG	TTG	GGC	AAA	AAA	ATC	AAG	GTA	TTT	1	2

CALW**EAQ**ELGKKIKVF																							
	TGT	GCC	TTG	TGG	GAG	G		CA				CAA	GAG	TTG	GGC	AAA	AAA	ATC	AAG	GTA	TTT	0	2
	TGT	GCC	TTG	TGG	GAG	G		CC				CAA	GAG	TTG	GGC	AAA	AAA	ATC	AAG	GTA	TTT	0	2
	TGT	GCC	TTG	TGG	GAG	G		CG				CAA	GAG	TTG	GGC	AAA	AAA	ATC	AAG	GTA	TTT	0	2
	TGT	GCC	TTG	TGG	GAG	G		CT				CAA	GAG	TTG	GGC	AAA	AAA	ATC	AAG	GTA	TTT	0	2

CALW**EVL**ELGKKIKVF																							
	TGT	GCC	TTG	TGG	GAG	GTG	C	T				A	GAG	TTG	GGC	AAA	AAA	ATC	AAG	GTA	TTT	1	1
	TGT	GCC	TTG	TGG	GAG	GTG	C	TG					GAG	TTG	GGC	AAA	AAA	ATC	AAG	GTA	TTT	1	2
	TGT	GCC	TTG	TGG	GAG	GTG	C	TT					GAG	TTG	GGC	AAA	AAA	ATC	AAG	GTA	TTT	1	2
	TGT	GCC	TTG	TGG	GAG	GTG	C	TC					GAG	TTG	GGC	AAA	AAA	ATC	AAG	GTA	TTT	1	2

CALW**EQ**ELGKKIKVF																							
	TGT	GCC	TTG	TGG	GAG							CAA	GAG	TTG	GGC	AAA	AAA	ATC	AAG	GTA	TTT	0	0
	TGT	GCC	TTG	TGG	GA			A				CAA	GAG	TTG	GGC	AAA	AAA	ATC	AAG	GTA	TTT	0	1

*Vγ9 and JγP gene segments are subject to nuclease activity, non-templated (N) nucleotide addition, and incorporation of palindromic (P) nucleotides, during recombination. Above are shown some of the possible different nucleotide sequences observed that generate the same CDR3 amino acid sequences, for five of the most common public Vγ9 sequences. N-nucleotides are shown in red and P-nucleotides are shown in blue*.

These features suggest the publicity of the Vγ9 repertoire is due to convergent recombination, a phenomenon proposed for generation of public TCRβ repertoires (24), whereby distinct recombination events “converge” to generate the same nucleotide sequences, and multiple nucleotide sequences “converge” to encode the same amino acid sequence. Venturi et al. proposed that public TCRβ responses arise from clonotypes with a high precursor frequency in two ways. Public sequences could arise independently multiple times in each individual by convergent recombination. Alternatively, precursor frequency could be increased if a single TCRβ rearrangement, which undergoes several rounds of proliferation after pre-TCR selection, could pair with many TCRα chains. Importantly, γδ T-cells do not undergo pre-TCR selection or proliferate after successful TCRγ rearrangement (but before TCRδ rearrangement) during T-cell development. Public Vγ9 sequences observed in adults must, therefore, result from convergent recombination.

High throughput Vδ2 TCR repertoire sequencing analyses provide corroborating evidence for convergent Vγ9 recombination. CDR3δ2 repertoires are more diverse than CDR3γ9 repertoires derived from Vγ9Vδ2^+^ T-cells from most adults (21, 23). Therefore, prevalent Vγ9 clonotypes (e.g., CALW**EVQ**ELGKKIKVF) do not reflect clonal expansion (if so equally large Vδ2 clonotypes would also be observed), but are likely recombined independently multiple times and pair with distinct Vδ2 chains. Single cell PCR in several individuals has substantiated the feasibility of this hypothesis, establishing unequivocally that public Vγ9 CDR3 clonotypes each paired with multiple Vδ2 clonotypes (23), confirming that public Vγ9 sequences arise frequently and independently. These findings prove that “convergent recombination” is an inherent feature of the Vγ9 repertoire, in keeping with public sequences exhibiting high precursor frequency because they have arisen *via* many independent recombination events in each donor. They also raise the question of whether, rather than requiring selective postnatal clonotypic expansion, the prevalence of public Vγ9 sequences may be preconfigured since birth.

## Shaping of the Adult Vγ9Vδ2 TCR Repertoire: Postnatal Selection

An intriguing question is whether Vγ9Vδ2^+^ T-cells expand *en masse* following microbial exposure during early childhood, concurrent with phenotypic maturation (4, 10), or whether dominant clonotypic selection operates, resulting in prevalent public Vγ9 clonotypes in adults (19). Of relevance, a recent study has compared adult peripheral blood with cord blood Vγ9Vδ2^+^ TCR repertoires (23). Importantly, the most prevalent public Vγ9 clonotype (CALW**EVQ**ELGKKIKVF) in the fetus (7) was also prevalent in cord (18, 23) and remains dominant in most adults (18, 20, 21). Moreover, other public Vγ9 clonotypes are frequently found in all these populations (16, 23). Also, the CDR3δ lengths in cord blood and adult peripheral blood are similar (23). Therefore, the public Vγ9 clonotypes present in adult peripheral blood Vγ9Vδ2^+^ T-cells are present at similar relative frequencies in cord blood Vγ9Vδ2^+^ T-cells. Furthermore, there were relatively subtle changes in the diversity of Vδ2-associated Vγ9 TCR repertoire from neonate to adult (23).

Despite these observations, postnatal changes in the Vδ2 repertoire are ultimately inconsistent with the concept of Vγ9Vδ2^+^ T-cell expansion *en masse*. Crucially, most Vγ9Vδ2^+^ cells in adult peripheral blood express Vδ2 recombined with Jδ1 (12), whereas in the cord blood most Vδ2 rearrangements use Jδ3, and to a lesser degree Jδ2 (12, 23) (Figure 1). This difference could be explained in two ways. One possibility is that extrathymic selection of specific clonotypes may occur in response to microbial exposure. Of relevance, it is currently unclear whether cord blood Vγ9Vδ2-Jδ3 cells are reactive to common pAg. While most Vδ2-Jδ1^+^ sequences in cord blood do generally contain a hydrophobic amino acid at position 5 (a motif previously linked to pAg reactivity) (23), fewer Vδ2-Jδ3^+^ sequences contain this motif (23). Consistent with this, Vγ9Vδ2^+^ T-cells from cord blood are generally less responsive to pAg than adult Vγ9Vδ2^+^ T-cells (10, 18, 25), however, the Vδ2 repertoire of responsive cells has not been reported, and conceivably only Vδ2-Jδ1 TCRs were responding in these assays.

A second possibility that could explain postnatal alterations in the Vδ2 TCR repertoire is a second wave of Vγ9Vδ2^+^ T-cell production after birth. Thymic Vγ9Vδ2^+^ T-cell output is thought to decrease after birth, based on failure to detect Vγ9 or Vδ2 gene expression in pediatric thymus samples (26), or detection of <10% of thymocytes expressing Vδ2 in thymi from children (4, 9). Surprisingly, Vγ9 expression was not detected in the thymus during childhood, despite its co-expression by Vδ1^+^ cells (21), which continue to be generated after birth (4, 26). Conceivably this issue warrants reinvestigation, and perhaps postnatal thymic Vγ9Vδ2^+^ T-cell generation has been underappreciated. Consistent with this, Ravens (22) and others (27, 28) have shown Vγ9Vδ2^+^ T-cell reconstitution following stem cell transplantation. Newly generated Vγ9Vδ2^+^ T-cells presumably originate in the recipient’s thymus (22). Detailed comparison of Vδ2-Jδ1 sequences in cord blood and adult repertoires (23) also hints at postnatal Vγ9Vδ2^+^ T-cell production. Although Vδ2-Jδ1 clonotypes are relatively uncommon in cord blood (most use Vδ2-Jδ3 at that time), those present often have shorter CDR3s, incorporating fewer N-nucleotides [as observed in fetal liver (5)] in comparison to the longer, more private Vδ2-Jδ1 clonotypes observed in adults. However, if the Vγ9Vδ2^+^ T-cells that predominate in adults are indeed generated in the postnatal thymus, we have observed no obvious differences in the Vγ9 repertoire of these cells, suggesting that the thymus continues to generate Vγ9-JγP rearrangements with low diversity even when TdT is expressed and when Vγ9 CDR3s found in Vδ1^+^ cells are highly diverse (21).

## Evidence for Prenatal Shaping of the Vγ9Vδ2^+^ TCR Repertoire

Postnatal processes clearly strongly influence the Vγ9Vδ2^+^ T-cell compartment. However, other events may also shape the prenatal Vγ9Vδ2^+^ repertoire (Figure 1). The Vγ9 repertoire is already highly restricted in CDR3 length during gestation, with public clonotypes evident (7), consistent with the cord blood Vγ9 repertoire (23). This indicates postnatal pAg exposure is not required for the selection of these features. However, the possibility that there might be some selection for pAg-reactive semi-invariant Vγ9Vδ2^+^ T cells before postnatal microbial exposure has been suggested previously (7), which potentially could operate intra- or extra-thymically. Conceivably, this could involve elevated levels of endogenous pAgs such as IPP derived from fetal isoprenoid metabolism, or pAg derived from placental microbiota; in addition, a specific selecting element, such as one or more of the BTN3 gene products could be involved (7). Bearing these possibilities in mind, enrichment of Jδ3 within cord blood Vδ2 sequences relative to adult peripheral blood could relate to more permissive positive selection of clonotypes responding to such fetal-specific selection events relative to postnatal responsiveness to exogenous microbially derived pAg. However, alternatively, genetic processes may explain the restricted nature of the Vγ9 repertoire in fetal and cord blood Vδ2^+^ cells. Consistent with this suggestion, the mouse OP9-DL1 thymic organ culture system can support Vγ9Vδ2^+^ T cell generation (9), arguing against a stringent positive selection step involving BTN3A1/pAg-mediated events. Of relevance to inherent genetic bias in Vγ9 chain recombination, whereas Vδ1-associated Vγ9 chains are diverse in length and rarely use JγP, Vδ2-associated Vγ9 CDR3 sequences are restricted in length, and exclusively utilize JγP, including in adults. These differences could merely reflect changes in gene segment accessibility during Vγ9Vδ2^+^ T-cell generation in early gestation, or regulation of Vγ9 chain recombination that favor simpler public Vγ9 rearrangements during the earlier timescale of fetal Vγ9Vδ2^+^ T-cell generation, before TdT is expressed (i.e., before 20 weeks of gestation) (29).

## Comparisons Between Vγ9Vδ2^+^ T-Cells and Semi-Invariant Mouse γδ T-Cell Subsets

Several features of the Vγ9Vδ2^+^ compartment suggest similarities to mouse γδ T-cell subsets (30). The early fetal wave of Vγ9Vδ2^+^ production, combined with the semi-invariant Vγ9Vδ2^+^ TCR repertoire, mirrors early waves of semi-invariant mouse γδ T-cells. The first T-cells to develop in mouse fetal thymus are Vγ5Vδ1^+^ dendritic epidermal T-cells, which have limited junctional diversity in both TCR chains (31). This is followed by production of Vγ6Vδ1 TCRs, also of limited diversity, then postnatal production of more diverse γδ T-cell populations using Vγ4, Vγ1, and Vγ7 chains (32). Some of these γδ populations undergo intrathymic or extrathymic selection events. DETC cells undergo intrathymic selection involving the BTN family member Skint1 (33, 34); the Vγ7 repertoire requires the presence of BTNL1/6 for extrathymic intestinal selection (35). Another semi-invariant mouse population expresses Vγ4 sequences of restricted length and diversity (analogous to public human Vγ9 sequences) with a germline-encoded Vδ5-Dδ2-Jδ1 sequence (36, 37), although its role and the signals that drive selection are unknown. The presence of γδ T-cells expressing semi-invariant TCRs in both mice and humans suggests this may reflect a shared paradigm for generation of T-cell populations with uniform reactivity to particular antigenic epitopes. Consistent with a related immunobiology, both BTN3A1 and BTN3A2/3 are important for Vγ9Vδ2^+^ T-cell recognition (38). However, while some semi-invariant mouse γδ T-cell populations can become hyporesponsive to TCR triggering following initial strong TCR signaling during development (39), this does not apparently apply to human Vγ9Vδ2^+^ T-cells. Notably Vγ9Vδ2^+^ T-cells remain responsive to both pAg and anti-CD3 stimulation, a feature which underlies their potential use in several cancer immunotherapy applications (40), and they also exhibit the potential for further TCR-mediated plasticity (41–44).

## Potential for Clonal Focusing in Response to Infectious/Stress Challenge

Although clear evidence supports a broad polyclonal Vγ9Vδ2^+^ T-cell response to pAg, the extent to which clonotype-specific responses occur remains unclear. Vγ9Vδ2^+^ T-cells expand in various infections (1) but TCR clonality is uncharacterized in most scenarios. While most healthy donors have similar Vγ9 repertoires composed of up to 80% public Vγ9 clonotypes and diverse Vδ2 clonotypes (23), a minority of healthy donors have one or several expanded Vγ9 and Vδ2 clonotypes reminiscent of Vδ1 expansions (21), with the top clone comprising 20–40% of all Vγ9 and Vδ2 CDR3s (23). These clones express Vγ9 clonotypes shared less frequently between adult donors, often with longer or more complex CDR3s containing more added N-nucleotides. In these donors, a Vδ2 clonotype of similar frequency is detected, and pairing of the top Vγ9 and Vδ2 clonotypes can be confirmed by single cell PCR. This clonal expansion correlated with a change in Vδ2^+^ T-cell phenotype to CD45RA^neg^CD27^neg^ (23), distinct from the CD45RA^neg^CD45RO^+^CD27^+^ phenotype observed in most healthy donors (45). The factors driving this clonal expansion and phenotypic maturation in these seemingly healthy donors are unclear. Ryan et al. (46) have also observed healthy donors with Vγ9Vδ2^+^ T-cells of differing effector phenotypes, although the clonality of Vγ9Vδ2^+^ T-cells was not examined. Expansion of particular Vδ2 clonotypes has also been noted in tuberculosis (47, 48), human leprosy (49), and in a macaque tuberculosis model (50). Public Vγ9 clonotypes were not shown to change during BCG infection in macaques (51), however, a lack of Vδ TCR clonotype data could have obscured the presence of clonotypic expansions with distinct Vδ2 chains. Conceivably clonal expansion may occur after Epstein–Barr virus or other common viral infections, and may underlie clonal expansions observed in otherwise healthy donors. Moreover, it is unclear how expansion of particular Vγ9Vδ2^+^ clonotypes helps protect the host, given the polyclonal response of Vγ9Vδ2^+^ T-cells to pAg. Conceivably expanded clones could respond with higher avidity, or alternatively could be reactive to different pathogen-specific stimuli, such as chemically diverse antigens. Additional work will no doubt address these questions.

## Conclusion

In summary, we suggest Vγ9Vδ2^+^ T-cell development is shaped by both prenatal and postnatal events (Figure 1), which impact TCR repertoire and pAg reactivity. Importantly, the human Vγ9Vδ2^+^ TCR repertoire is composed of highly public Vγ9 chains produced by frequent recombination events that occur in every individual, resulting in a semi-invariant repertoire largely preconfigured from birth for pAg reactivity. These Vγ9 chains may undergo prenatal selection based on pAg reactivity, or unknown factors may constrain Vγ9-JγP rearrangements. Alongside public Vγ9 sequences, the Vδ2 repertoire is very diverse and private, and changes between neonatal and adult Vδ2 TCR repertoires suggest several selection events throughout life. Vδ2-Jδ3 TCRs are prevalent in cord blood and these may be positively selected in fetal development for recognition of host pAgs, or these rearrangements may be preferentially generated in early gestation. Vδ2-Jδ1 chains with longer CDR3 and hydrophobic amino acids at position 5 ultimately dominate the Vδ2 repertoire in adults, and these may be selected from rare rearrangements in cord blood following microbial pAg exposure, or further Vγ9Vδ2^+^ T-cell generation may occur in the postnatal thymus. Nevertheless, these selection events produce a repertoire that exploits the somatically recombined Vγ9Vδ2^+^ TCR as a surrogate pattern recognition receptor to sense pAgs. Further clonal selection appears to occur in some healthy adults and during some infections, however, exactly what protection such favored clonotypes provide that are not provided already by the broad Vγ9Vδ2^+^ TCR repertoire is an intriguing question future studies can address.

## Author Contributions

CW, MD, and BW jointly conceived the concepts presented in this review. CW analyzed data, prepared figures, and wrote the first draft; MD prepared figures and helped finalize the manuscript; BW helped plan and write the final manuscript.

## Conflict of Interest Statement

The authors declare that the research was conducted in the absence of any commercial or financial relationships that could be construed as a potential conflict of interest.
